# Abnormal Activity in the Precuneus during Time Perception in Parkinson's Disease: An fMRI Study

**DOI:** 10.1371/journal.pone.0029635

**Published:** 2012-01-06

**Authors:** Petr Dušek, Robert Jech, Tomáš Sieger, Josef Vymazal, Evžen Růžička, Jiří Wackermann, Karsten Mueller

**Affiliations:** 1 Department of Neurology and Center of Clinical Neuroscience, Charles University in Prague, 1st Faculty of Medicine and General University Hospital, Prague, Czech Republic; 2 Department of Cybernetics, Czech Technical University in Prague, Faculty of Electrical Engineering, Prague, Czech Republic; 3 Department of Radiology, Na Homolce Hospital, Prague, Czech Republic; 4 Department of Empirical and Analytical Psychophysics, Institute for Frontier Areas of Psychology and Mental Health, Freiburg im Breisgau, Germany; 5 Max Planck Institute for Human Cognitive and Brain Sciences, Leipzig, Germany; University Medical Center Groningen UMCG, Netherlands

## Abstract

**Background:**

Parkinson's disease (PD) patients are deficient in time estimation. This deficit improves after dopamine (DA) treatment and it has been associated with decreased internal timekeeper speed, disruption of executive function and memory retrieval dysfunction.

**Methodology/Findings:**

The aim of the present study was to explore the neurophysiologic correlates of this deficit. We performed functional magnetic resonance imaging on twelve PD patients while they were performing a time reproduction task (TRT). The TRT consisted of an encoding phase (during which visual stimuli of durations from 5s to 16.6s, varied at 8 levels were presented) and a reproduction phase (during which interval durations were reproduced by a button pressing). Patients were scanned twice, once while on their DA medication (ON condition) and once after medication withdrawal (OFF condition). Differences in Blood-Oxygenation-Level-Dependent (BOLD) signal in ON and OFF conditions were evaluated. The time course of activation in the brain areas with different BOLD signal was plotted. There were no significant differences in the behavioral results, but a trend toward overestimation of intervals ≤11.9s and underestimation of intervals ≥14.1s in the OFF condition (p<0.088). During the reproduction phase, higher activation in the precuneus was found in the ON condition (p<0.05 corrected). Time course was plotted separately for long (≥14.1s) and short (≤11.9s) intervals. Results showed that there was a significant difference only in long intervals, when activity gradually decreased in the OFF, but remained stable in the ON condition. This difference in precuneus activation was not found during random button presses in a control task.

**Conclusions/Significance:**

Our results show that differences in precuneus activation during retrieval of a remembered duration may underlie some aspects of time perception deficit in PD patients. We suggest that DA medication may allow compensatory activation in the precuneus, which results in a more accurate retrieval of remembered interval duration.

## Introduction

Parkinson's disease (PD) patients are deficient in tasks requiring time estimation. This deficit is partially improved by dopamine (DA) medication. Noted among these time estimation tasks were: prospective time production task [1], retrospective time estimation task, time reproduction task [2] and time discrimination task [3]. It was shown that PD patients give longer prospective and shorter retrospective time estimates and the results were explained in terms of slower internal timekeeper speed [2]. The slower internal timekeeper rate hypothesis is in accordance with animal research, which has shown that DA agonists increase and antagonists decrease the rate of the hypothetical “internal clock” [4]. However, time perception deficits accountable to different timekeeper rate were not replicated by other studies [5]–[8] and the nature and relevance of timing deficits in PD remain a rather controversial topic. The role of basal ganglia [9], [10] and DA transmission [11] in time perception has also been questioned recently.

A specific timing disorder has been described in PD patients, which is characterized by a tendency to overestimate shorter and underestimate longer interval, when estimated within one experimental session. In the original study, PD patients reproduced an 8 second interval as 8.9 seconds long and a 21 second interval as 17.3 seconds long [12]. This phenomenon was named the migration effect and was explained in terms of abnormal memory retrieval. Underestimation of the longer interval did not occur when it was learned and reproduced alone. This migration effect appears only after antiparkinsonian medication withdrawal (OFF condition). Improvement has been observed after DA replacement therapy (ON condition) [12] and even after deep brain stimulation of the subthalamic nucleus (DBS-STN) [13].

The neural basis of PD patients' timing deficit is still poorly understood. One study reported the migration effect to be more pronounced in left hemi-Parkinsonian patients [14]. This is in agreement with the supposed prominent role of the right hemisphere in time perception [15], [16]. Timing deficit in PD is improved by repetitive transcranial magnetic stimulation (rTMS) of the right dorsolateral prefrontal cortex (DLPFC) [17], suggesting that dysfunctional mnemonic and decisional processes subserved by this brain area contribute to disordered timing. The basal ganglia, cerebellum, DLPFC, supplementary motor area (SMA), insular and parietal cortex have been suggested to play a role in time perception (for review see [18], [19]). It has also been suggested that brain areas activated during the processing of sub-second and supra-second intervals are not identical. Whereas sub-second intervals seem to be processed by a motor circuitry comprised of the primary sensorimotor cortex, SMA and cerebellum; supra-second intervals more likely activate the associative cognitive areas including DLPFC, insular and parietal cortices [20]. Recent voxel-wise meta-analysis stressed the importance of SMA and right inferior frontal gyrus for time perception across various perceptual and motor timing tasks including sub-second and supra-second intervals [21]. Many of the above mentioned areas are DA dependent and have been shown to be dysfunctional in PD [22], [23]. However, little is known about whether and how these dysfunctions contribute to PD patients' deficit in time perception.

So far, several neuroimaging studies investigated the neurophysiologic basis of timing deficit in PD patients [24]–[27]. However, these studies examined motor timing of relatively short durations, which makes it difficult to distinguish between timing related and motor related activity. Another study examined time perception in PD using time interval comparison tasks with durations 1.2 and 1.8 seconds [28]. Different brain areas were found to underlie the PD patients' deficits during the encoding and decision phases. Striatal dysfunction was found during both phases, but working memory network comprising of DLPFC, parietal cortex and cerebellum was only deranged during encoding, while posterior cingulate and parahippocampal cortices only during decision phase.

As yet, no studies have examined brain activation in PD patients during the perception of longer supra-second intervals. The human subjective perception of time flow supposedly ranges from 3 to dozens of seconds [29]. This time range is also where the migration effect has occurred in previous studies [12], [13]. The aim of our study was to elucidate the neurophysiologic basis of PD patients' deficit in time perception in the OFF condition, namely of the migration effect, using functional magnetic resonance imaging (fMRI). We hypothesized to find differences in the cognitive, rather than in the motor network, since estimation of supra-second intervals is dependent on attentional, working memory and decisional processes. We also expected greater differences in brain activation patterns during the reproduction phase, since the migration effect was shown to be a result of memory retrieval dysfunction.

## Methods

### Ethics statement

A signed, informed consent was obtained from all participants and the study was approved by the ethics committee of General University Hospital in Prague.

### PD patients

Twelve mild to moderate PD patients, 10 male, age 60.0 (mean)±8.7 (SD) years, disease duration 6.8±3.2 years, 6 with dominant right and 6 with dominant left hemi-body involvement, participated in the study. Only right-handed subjects without signs of dementia (Mini-Mental State Examination score ≥28) or severe depression (Beck Depression Inventory score ≤18) were included. The degree of motor impairment was assessed by the motor subscale of Unified Parkinson's disease rating scale (UPDRS-III). Patients with a tremor as a dominant symptom and patients with substantial dyskinesias were excluded. All but one patient were on a stable combined DA medication with levodopa and DA agonist, taking 680±321 mg of levodopa equivalent [30]. One patient was treated with amantadine only and was given a single dose of levodopa (250 mg) in the ON condition after domperidone pre-treatment.

Each patient was examined twice, in ON and OFF conditions, in a counter-balanced order. The scanning was always performed at the same time of day and the interval between fMRI sessions ranged from 1 to 5 weeks. The examination in the OFF condition was performed after 78 hours DA agonist and 12 hours levodopa withdrawal. In the ON condition, patients were taking their regular antiparkinsonian medication and were scanned 1 hour after a DA medication dose, so they were studied in their best ON condition. UPDRS-III was examined before scanning and the score was 24±7 in the OFF condition and 16±8 in the ON condition (paired t-test, p<0.001). A simple reaction time task using a visual stimulus (blue diamond displayed for 100 ms) and a random inter-stimulus interval in the range 2–5 seconds was employed in order to examine potential effects of motor slowing on TRT.

### Time reproduction task

The same paradigm as in our previous study [31] was used. TRT consisted of two phases: encoding and reproduction. During the encoding phase, subjects had to retain the duration of a presented visual stimulus – a gray square with a red cross in the centre. The encoding phase was followed by a constant inter-stimulus interval (10 s), during which an indifferent stimulus (gray cross) was displayed. In the reproduction phase, the subject had to reproduce the retained duration by pressing a joystick button with his/her right index finger. The reproduction phase started by presenting a gray square with a green cross in the centre, which was displayed for 1.5-times the duration of the encoding interval. If the subject failed to press the button by the end of the reproduction phase, the trial was considered to be a missed response and was not used for the analysis. Each experimental session comprised 16 trials. In each trial, the duration of the encoding interval was selected pseudo-randomly out of a set of eight durations: 5.00, 5.95, 7.07, 8.41, 10.00, 11.89, 14.14 and 16.82 s (i.e., a geometric sequence x_i+1_ = x_i_.2^1/4^) so that each interval was used twice. Subjects were instructed to fixate centrally the cross throughout the task and to avoid mental counting.

Patients practiced the task off the scanner before each session. For training purposes, TRT with time intervals of 5, 10 and 16.82 seconds was used. Only behavioral data from the scanning session were used for analysis.

In order to control for movement planning and execution we performed a control task, where patients were required to produce random button presses. In this random button-press task (RBPT), which was performed during the same session as TRT and lasted 4 minutes and 50 seconds, patients were instructed to press a joystick button randomly several times during the task whenever they wanted.

### FMRI image acquisition

Blood Oxygenation Level Dependent (BOLD)-fMRI was performed on a Siemens Symphony 1.5T scanner, using the gradient echo echo-planar sequence (TR = 2.9 s, TE = 54 ms, FA = 90 deg). In the TRT, a total of 260 volumes consisting of 25 continuous, 4-mm thick axial slices using an in-plane resolution of 1.8×1.8 mm^2^ (image matrix 88×128 obtained with zero-filling) was acquired. In the RBPT, 100 volumes using the same parameters were acquired. During the ON condition, a structural image was acquired using a 3D T1- weighted magnetization-prepared rapid gradient echo acquisition (MP-RAGE) sequence with an in-plane resolution of 0.64×0.64 mm^2^ (data matrix 512×512), 160 slices with a slice thickness of 1.65 mm were obtained.

### fMRI image preprocessing and statistical analysis

The data analysis was performed twice using SPM8 (Statistical Parametric Mapping, Wellcome Trust Centre for Neuroimaging, London, UK) [32] and Lipsia (Leipzig Image Processing and Statistical Inference Algorithms, Max Planck Institute for Human Cognitive and Brain Sciences, Leipzig, Germany) [33]. The data was evaluated using both packages in order to ensure the correctness of the results by two analytical approaches. Post-hoc testing was done solely using Lipsia.

Functional data was corrected for motion and differences in slice acquisition time. Hereafter, functional slices were aligned with a 3D stereotactic coordinate reference system using a rigid linear registration. The registration parameters were acquired to achieve an optimal match (maximum mutual information) between the functional slices and a 3D reference data set. In SPM8, the functional slices were aligned with the MNI152 template (Montreal Neurological Institute) [34], and in Lipsia, the data was standardized to the Talairach stereotactic space [35]. After normalization, the data was smoothed over with a Gaussian filter of 7 mm FWHM. We also used a temporal high-pass filter with a cut-off frequency of 1/90 Hz.

The statistical evaluation was based on a least-squares estimation using the general linear model for serially autocorrelated observations [36]–[38]. The design matrix was generated with a synthetic hemodynamic response function [39], [40]. The design was generated using the two experimental conditions, encoding and reproduction. The encoding phase was defined by the time how long the red cross appeared on the screen. The reproduction phase was defined as the interval starting with the appearance of the green cross and ending with the subjects' button press. For both experimental conditions, contrast images were generated using the associated parameter estimates. Because all patients were measured twice in the ON and OFF conditions, four contrast images were computed for each patient. The contrast images were put into two second-level analyses using paired t-tests in order to search for differences between the ON and OFF conditions during encoding and reproduction phases. We further compared the activation preceding the button press in TRT and RBPT with SPM8 using a parameter estimate looking from 8-to-3 seconds before button press. We analyzed the data using a flexible-factorial-design with the factors of task (RBPT/TRT) and treatment condition (OFF/ON), taking the individual variability into account.

The results of the second-level-analyses were corrected for multiple comparisons. In SPM8, we used a family-wise error (FWE) correction. In Lipsia, the results were corrected using cluster-size and cluster-value thresholds obtained by Monte-Carlo simulations (clusters in the resulting maps were obtained using an initial threshold of p<0.005). These corrected maps were used to define regions of interest (ROIs) in order to analyze trial averages of the (pre-processed) functional data.

## Results

### Behavioral results

A repeated measures ANOVA with the factors of treatment condition (ON, OFF) and interval (5.00, 5.95, 7.07, 8.41, 10.00, 11.89, 14.14 and 16.82 seconds) was used for behavioral data analysis.

**Figure 1:**
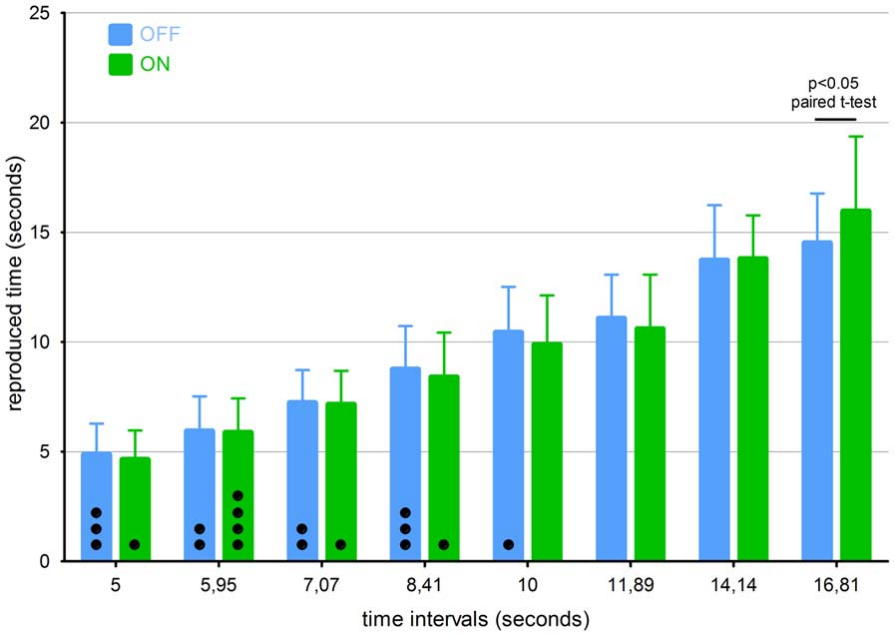
Mean interval reproductions. Mean reproduced durations ± SD for all subjects in the OFF (blue) and ON (green) conditions. Direct comparison between the ON and OFF condition was significant only for the 16.8 s interval (paired t-test, p<0.05). Dots inside of the bars represent missed responses for each interval and condition.

The main effect of treatment condition was not significant, but there was a trend towards significant interaction between condition and interval (F (7, 77) = 1.9, p = 0.088). There were 11 missed responses in the OFF condition and 7 in the ON condition. When compared to the ON condition, the reproduction of intervals ≤11.9 seconds were overestimated and intervals ≥14.1 seconds were underestimated in the OFF condition (Figure 1). Separate *post-hoc* comparisons of individual time intervals showed a significant difference only at the longest (16.8 s) interval, where the reproduced time was significantly longer in the ON condition (paired t-test, p<0.05 uncorrected).

There were no significant differences in reaction times between the OFF and ON conditions (257±50 ms vs 261±58 ms).

### Imaging results

**Figure 2:**
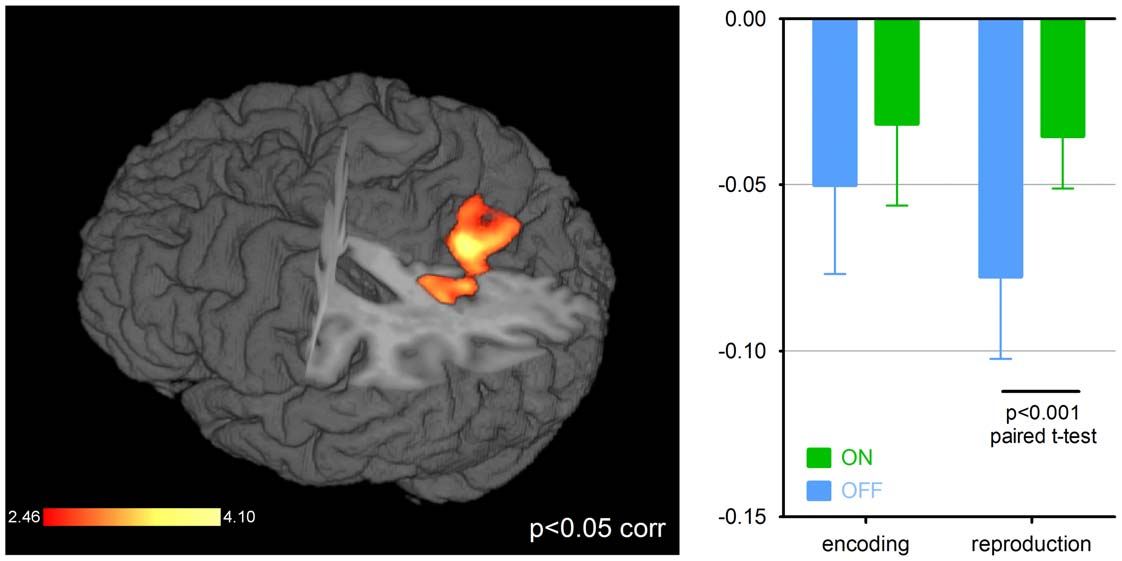
Area activated in the reproduction phase, ON>OFF. On the left, results of the second-level group analysis (p<0.05 corrected) are rendered to a structural MRI image using vlrender function from Lipsia package. On the right, the mean contrast estimate plots ± SD in the right precuneus (coordinates 4 −62 26) during encoding (left) and reproduction (right) phases. There was deactivation of the precuneus found in all four conditions (encoding ON, encoding OFF, reproduction ON, reproduction OFF). Comparison between OFF (blue) and ON (green) conditions was significant only during the reproduction phase (paired t-test, p<0.001), when deactivation was more marked in the OFF condition.

The software packages SPM8 and Lipsia gave the same results: In the encoding phase, both software packages did not show any significant differences between ON and OFF conditions. In the reproduction phase, the BOLD signal in the bilateral precuneus was significantly higher in the ON compared to the OFF condition (p<0.05 FWE-corrected on the cluster level, see Figure 2, Table 1).

**Table 1:** fMRI group results of the reproduction phase (computed in Lipsia).

region	BA	coordinates(x, y, z)			volume(mm^3^)	Z score (max)	p value
**ON>OFF**							
Left precuneus	7	−8	−66	38	320	−3.67	0.001*
Right precuneus	31	4	−62	26	280	−3.55	0.003*

*significant at p<0.05 corrected.

There was not such a difference in activation during the time period preceding the button press in the RBPT. While comparing TRT and RBPT we found a significant interaction between factors task and treatment condition in the precuneus bilaterally (p<0.001, FWE-corrected on the cluster-level), which indicates that the difference in precuneus activation is related specifically to the TRT and not to the button press preparation and execution.

**Figure 3:**
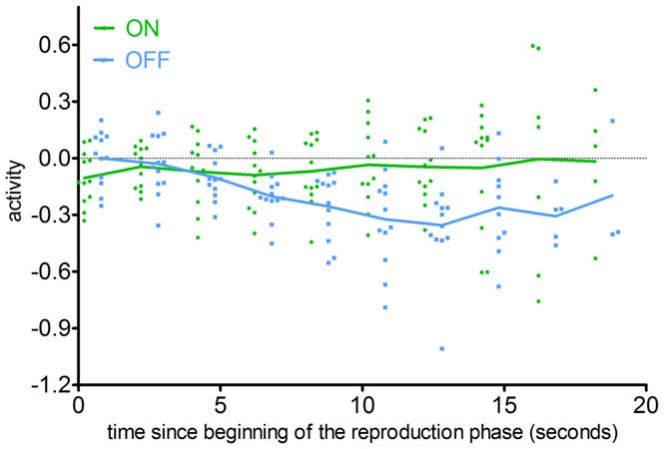
Time-course plot of the activity in precuneus (coordinates 4 −62 26) during the reproduction phase. Each dot represents mean activity in a time point for all trials of one subject in OFF (blue) and ON (green) conditions. Lines connect group means for all subjects. For better clarity, dots are jittered.

The activity in the precuneus was *post-hoc* analyzed with a focus on comparing activation during encoding and reproduction phases, time course of activation and differences between short and long durations. Contrast estimate plots in the precuneus showed that the BOLD signal decreased in both the ON and OFF conditions and during the encoding and reproduction phases. A significant difference was observed during reproduction phase only, due to larger deactivation in the OFF condition (paired t-test, p<0.001, Figure 2). The time course plot of precuneus activity during the reproduction phase superimposed for all reproduced intervals showed that in the ON condition, the BOLD signal remained largely constant, whereas in the OFF condition it gradually decreased to approximately 10 seconds and then remained constant (Figure 3).

**Figure 4:**
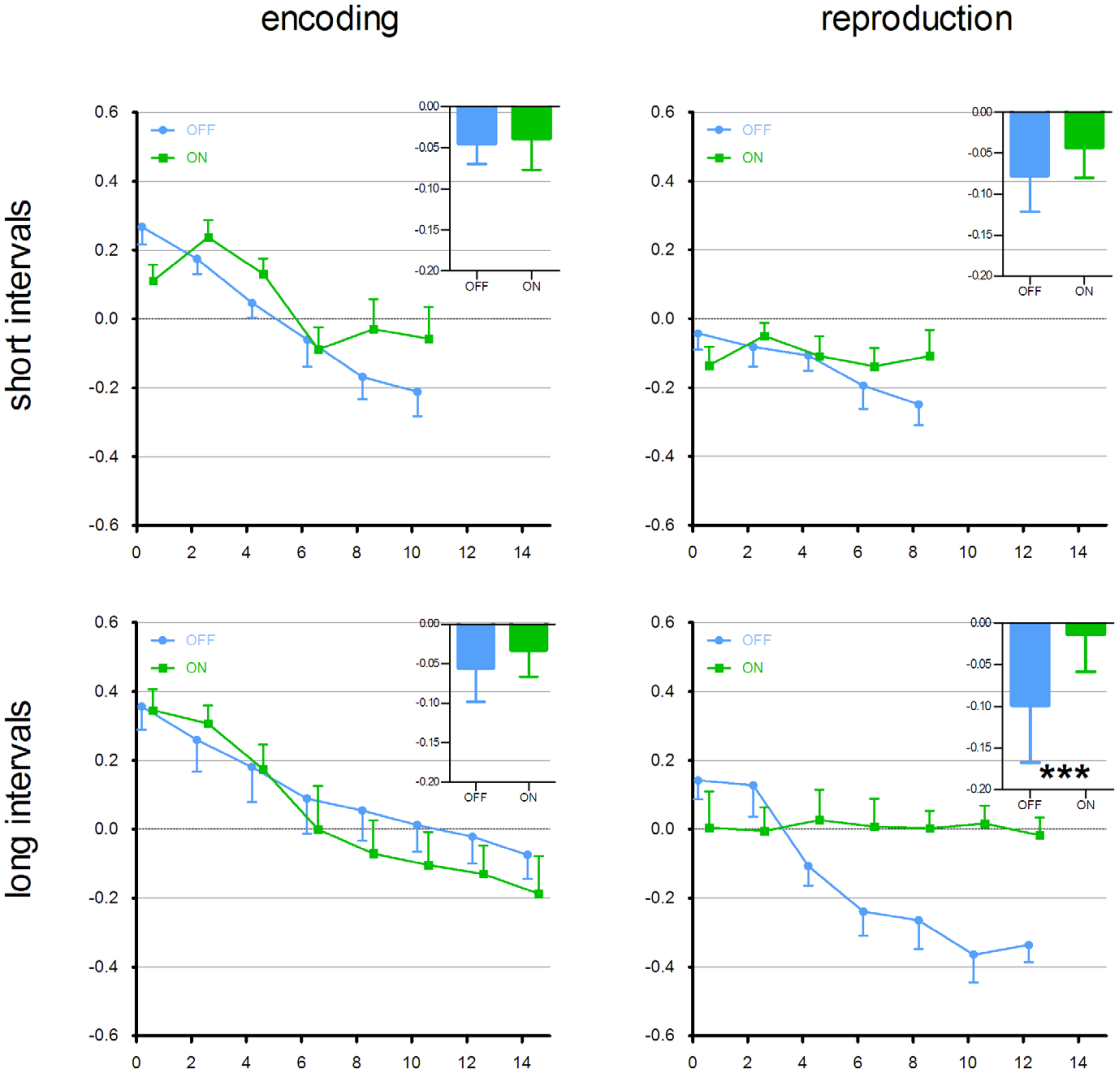
Time-course plots of the activity in the precuneus (coordinates 4 −62 26) according to the phase and interval duration. Activity is plotted during encoding short (upper left), encoding long (lower left), reproduction short (upper right) and reproduction long (lower right) conditions. Group means ± standard error of mean (SEM) for OFF (blue) and ON (green) conditions are depicted. Time points, where at least 3 observations per subject and 6 subjects per time point were not available, were excluded. Therefore encoding of short intervals was followed up to 10 seconds, encoding of long intervals up to 14 seconds, reproduction of short intervals up to 8 seconds and reproduction of long intervals up to 12 seconds. Bars in the upper right corner of each graph represent overall contrast estimate mean ± SD for each condition. The comparison between OFF and ON conditions yielded significant differences only for the reproduction of long intervals (marked with ***, paired t-test p<0.001).

We were also interested whether there is any difference between precuneus activity during short (overestimated) and long (underestimated) time intervals. The time course plots for short (5.00, 5.95, 7.07, 8.41, 10.00 and 11.89 seconds) and long (14.14 and 16.82 seconds) intervals during encoding and reproduction phases were computed. During the encoding phase, there was a gradual decrease in the BOLD-signal in all conditions. During reproduction phase, there was a significant difference between the ON and OFF conditions only in long intervals (paired t-test, p<0.001). In the OFF condition, there was a gradual reduction of the BOLD-signal, whereas in the ON condition, activity remained constant. This difference was not seen in short intervals (Figure 4).

## Discussion

In this study, we sought for differences in brain activation during time perception in PD patients ON and OFF their DA medication using fMRI. There were no differences in brain activation during the encoding phase of TRT, but during the reproduction phase there was significantly larger deactivation in the precuneus bilaterally in the OFF condition. The time-course analysis showed a gradual decrease of activation in the precuneus during encoding in ON and OFF conditions and during reproduction in the OFF condition. This pattern of precuneus activation was not observed during the reproduction phase in the ON condition when the course of its activation was flat. This effect became particularly apparent during the long intervals.

The migration effect in PD cannot be explained in terms of decreased internal timekeeper speed [2], [3], [32], [41] but may rather arise from mutual interaction and averaging among memory traces of time intervals during their retrieval [42]. Another hypothetical reason is an abnormal, non-linear “time-accumulator” [43]. Even though, there was only a trend in the interaction between condition and interval, our behavioral results exhibit migration effect properties with a cut-off between 11.9 and 14.1 seconds. In the OFF condition, compared to the ON condition, reproductions of intervals ≤11.9 seconds were overestimated and ≥14.1 seconds were underestimated. All previously performed studies on PD patients have found a migration effect in 2 intervals, one which was shorter and the second which was longer than the cut-off found in our study [12]–[14], [42]. It was shown that underestimation of the longer interval did not occur when it was reproduced as a single time interval and therefore it is unlikely that overestimation of the shorter and underestimation of the longer interval reflect dysfunctions of two different timing systems. It would be interesting to investigate whether the migration effect in PD patients also occurs in the intervals below and above this cut-off. A study looking for migration effect in intervals of 0.5 and 2 seconds found only abnormal reproduction of the 2 seconds interval [44]. This finding supports the notion that perception of sub-second intervals is mediated by a different brain network which is DA independent.

Interestingly, an averaging tendency in time interval reproductions has been shown also in healthy subjects [45]. A recent study showed that the “migration effect” exists in healthy subjects during the reproduction of sub-second intervals. The same interval was reproduced differently when presented in the context of intervals with different durations. Reproduced intervals exhibited a systematic tendency to drift towards the mean of the prior distribution from which they were drawn. The authors concluded that subjects rely more on the prior mean of remembered durations when measurements are less reliable, e.g. in the case of attention deficit or in more difficult tasks [46]. Both of the latter are likely the case of PD patients in the OFF condition. Interestingly, healthy subjects exhibited the migration effect when the time reproduction task was made more difficult by involving a more demanding motor response and by prolonging the delay between encoding and reproduction [47].

Surprisingly, we didn't observe differences between ON and OFF conditions in brain areas previously connected to interval timing [18], [19] and the only difference was reduced deactivation of the precuneus during the reproduction phase in the ON condition. There are several questions arising from this finding. First, whether differences in precuneus activation are directly linked to timing or reflect unspecific effect of DA medication on brain activation patterns? And second, how are these changes related to the migration effect?

The precuneus has previously been connected to cognition and treatment effects in PD. It is a part of the postulated PD-related cognitive network, which is comprised of brain areas with a correlation between metabolism and cognitive functions in PD [23], [48]. The degree of hypometabolism in the precuneus correlated with impaired performance of memory and executive tests in PD. Increased precuneus activity has been also found after levodopa administration and DBS-STN, which was explained by increased excitatory thalamo-cortical activity rather than by a direct modulation [49], [50]. Moreover, DA transmission enhancers, such as cocaine, were shown to directly influence neurovascular coupling in experimental animals [51], which may be another possible confounding factor. Nevertheless, our result of the relatively increased BOLD response in the precuneus in the ON condition is not merely an unspecific effect of DA medication, since it occurs only during the reproduction phase of TRT, but not during the encoding phase or during the RBPT and hence is task specific.

The precuneus has not convincingly been linked to interval timing, however, we observed a gradual BOLD-signal decrease in the precuneus which correlated with the time interval duration in our previous study using the same task design in healthy subjects [31]. A study using an interval discrimination task of intervals between 1–2 seconds in healthy subjects showed that precuneus activity correlated with memory distortions during encoding [52]. An increased activity of default brain network was also found as a substrate of subjective time dilatation, i.e. overestimation, which was described when subjects judged the duration of looming objects compared to steady objects with the same duration [53], [54].

Precuneus activation is consistently reported in tasks with mental imagery, self-processing and autobiographical memory retrieval [55], [56]. The precuneus is also a principal part of the so-called default brain network (DBN), which is connected to self-referential mental activity during rest and its activity is decreased during externally goal-directed activity [57], [58]. The magnitude of its deactivation was found to be proportional to task demands and task performance [59]–[62]. Several studies documented the relation between DA transmission and precuneus activity. Increased dopamine transporter (DAT) concentration in the striatum, causing decreased DA effects due to enhanced re-uptake, was shown to reduce the deactivation of the precuneus during a visual attention task [63]. On the other hand, homozygotes of val/val catechol-O-methyl transferase (COMT) polymorphism with lower synaptic DA levels exhibited a greater deactivation of the precuneus and posterior cingulate cortex (PCC) while performing a working memory task [64].

Previous studies have shown conflicting results regarding dysfunction of DBN in PD and its modulation by DA therapy. Results of two studies suggest reversed pattern of DBN behavior, i.e. activation during an executive task and deactivation during a control task in DA-depleted PD patients, which is normalized after levodopa intake. These studies also showed dissociation between the anterior and posterior parts of DBN, since changes in activity were pronounced in the precuneus/PCC while the medial prefrontal cortex was relatively unaffected [65], [66]. Moreover, inability to deactivate DBN was associated with a worse performance in the Montreal card sorting task, which is a cognitive task with high requirements on executive functions [66]. On the other hand, Argyelan et al. found that the effects of levodopa on DBN activity differs according to baseline task performance. In PD patients with poor baseline performance, levodopa increased precuneus activity, while improving the performance of the sequence learning task. The opposite was true for PD patients with good baseline performance, where levodopa reduced precuneus activation and led to a loss of medial prefrontal cortex deactivation [67]. In line with their findings, we found a trend to better performance, i.e. amelioration of migration effect, in the ON condition, while deactivation response in precuneus was reduced.

The gradual decrease of precuneus activity observed in our study during the encoding phase regardless of the interval duration or medication condition may thus reflect gradual DBN deactivation with an increasing memory load as the duration is being remembered. It is not clear, whether the higher activity of the precuneus during reproduction of long intervals in the ON condition is a compensatory activity or an aberrant activity coming from the inability to suppress DBN. Several factors favor the compensatory hypothesis. First, task performance tended to improve in the ON condition. Second, there is no general dysfunction of DBN in the ON condition, since it is capable of normal deactivation during the encoding phase. Third, migration effect arises from dysfunctional retrieval phase of TRT and the precuneus has a role in episodic memory retrieval [55]. Our behavioral data suggest that the perception of longer intervals is more distorted when compared to shorter intervals and that the change in precuneus activity also occurs only during the reproduction of long intervals. It is likely that longer intervals need more episodic memory resources for retrieval and thus higher activity in the precuneus may reflect increased need for memory retrieval capacity. We can speculate that besides averaging, which seems to be the brain default strategy when attention and working memory processes are not working reliably, memory retrieval dysfunction may contribute to underestimation of longer intervals. Memory of longer intervals is prone to decay [68] and the consequent retrieval of less information from the memory may lead to time interval underestimation proportional to the duration of the reproduced interval. Increased precuneus activity in the ON condition may reflect compensatory activity for the memory retrieval deficit. Our results indicate that abnormal performance in timing tasks in PD patients is not due to dysfunction of internal clock but due to dysfunctional retrieval and that the positive effect of DA medication on a cognitive timing may be related to a relative increase of activity in the precuneus. Our results together with previous studies [63]–[67] suggest that DA transmission may increase or decrease precuneus activity during cognitive task depending on task characteristics, baseline performance and also possibly on PD stage.

It is important to mention also several limitations of our study. Estimation of long supra-second intervals includes multiple cognitive functions and is subject of poorly controllable confounds such as attention fluctuations, habituation or subconscious usage of environmental cues. This results in very high within-subject and between-subjects variability of temporal estimates which is proportional to the length of the interval. Due to noise in the results it is not possible to correlate individual behavioral results with the specific BOLD effect. While it is impossible to directly correlate neural activity with the behavioral results, we cannot exclude the possibility that the group difference in precuneus activation reflects other processes than timing and memory retrieval, such as distinctive habituation or attention decay in the ON and OFF conditions. Due to same reasons, it is not possible to plot reliably the time-course of activity in precuneus beyond 8 seconds in the short intervals and beyond 12 seconds in the long intervals (Figure 4). Lastly, although we excluded demented patients by Mini-Mental State Examination, we didn't perform detailed neuropsychological examination or any other tests for memory encoding and retrieval functions in ON and OFF condition. Thus, we cannot further support our speculation about improved memory retrieval function in the ON condition.
